# Dyspnœa from Bronchocele Relieved by Division of the Cervical Fascia

**Published:** 1858-01

**Authors:** 


*Dyspnœa from bronchocele relieved by division of the cervical fascia.*—M. Cusack relates (Dub. Hosp. Gaz.) a case of bronchocele in a boy, æt. 17 years, which, by its pressure, caused such a degree of suffocation as to threaten his life. He obtained some relief by making pressure upon the tumor, thus relaxing the fascia. His symptoms finally became so alarming that surgical interference was necessary, when Mr. C. determined to divide the cervical fascia over the tumor. Before the operation was completed, respiration ceased, and he was obliged to open the trachea, and resort to artificial respiration. The patient revived; the wound was closed, and the relief was permanent; due, as the operator believes, to division of the fascia.—*Ib.*

